# CAR trafficking does matter: *prospects of ‘Chimeric antigen receptor designed to prevent ubiquitination and downregulation showed durable antitumor efficacy’*

**DOI:** 10.1093/jmcb/mjaa045

**Published:** 2020-09-16

**Authors:** Wentao Li, Han Yang, Li Li, Haopeng Wang

**Affiliations:** 1 School of Life Science and Technology, ShanghaiTech University, Shanghai 201210, China; 2 Shanghai Institute of Biochemistry and Cell Biology, Center for Excellence in Molecular Cell Science, Chinese Academy of Sciences, Shanghai 200031, China; 3 University of Chinese Academy of Sciences, Beijing 100049, China

Chimeric antigen receptor (CAR) is a synthetic antigen receptor containing a specific antigen-recognition ectodomain to make T cells selectively attack cancer cells, a hinge-transmembrane region to confer stable surface expression, and one or more intracellular signaling domains to regulate T-cell activation. CAR T-cell therapy has produced unprecedented clinical outcomes for treating cancers, particularly B-cell malignancies. However, increasing clinic data reveal some limitations of current CAR T therapies. For example, >30% of B-cell malignancy patients who initially achieved complete remission encountered relapses after 1-year infusion of CAR T cells. In case of solid tumors, most of the patients did not benefit from CAR T treatment (Park et al., 2018; Schmidts and Maus, 2018). CAR T-cell persistence, defined as how long CAR T cells could survive *in vivo* after infusion into patients, is one of the major factors affecting the clinical outcomes of CAR T therapy (Porter et al., 2015). Therefore, it is important to understand the molecular mechanism(s) controlling the persistence of CAR T cells. 

In T cells, T cell receptor trafficking is dynamically regulated and intensively investigated. In contrast, how CAR trafficking is regulated in CAR T cells remains largely unknown. Recently, we showed that, in resting CAR T cells, CAR surface expression is stable and CAR is constitutively internalized and recycled back to the cell surface (Li et al., 2020). However, the engagement of tumor antigens triggers rapid CAR ubiquitination, which targets internalized CAR for lysosomal degradation, leading to CAR downmodulation. To block CAR ubiquitination, we mutated all lysine residues of CAR intracellular domains to arginine (CAR^KR^), which prevents CAR from lysosomal degradation and promotes recycling of CAR back to the cell surface. We named this new CAR as ‘recyclable CAR’.

Compared to wild-type (WT) CAR, recyclable CAR has several unique features (see Table 1). First, due to the blockade of CAR degradation, the half-life of recyclable CAR protein is substantially increased. This feature could potentially enhance the efficacy of some CAR therapy strategies. For example, the *in vitro*-transcribed mRNA approach is frequently used in CAR T therapy development (a.k.a. RNA CAR) because of its inherent safety. However, the efficacy of RNA CAR is often limited by the short half-life of mRNAs. Recyclable CAR may offer a potential solution to optimize pharmacokinetic properties of mRNA CAR. Second, the recyclable CAR T cells sustain surface CAR expression following the engagement of tumor antigens, which likely contributes to more effective long-term tumor-killing ability. Last but not least, with the incorporation of the 4-1BB co-stimulatory domain, the recyclable CAR design promotes T-cell *in vivo* persistence. We compared *in vivo* persistence of WT 4-1BB-CAR T cells and recyclable 4-1BB-CAR T cells by injecting the same number of CAR T cells to tumor-bearing mice. Surprisingly, the recyclable CAR T cells displayed greater persistence (>100-fold more CAR^KR^ T cells than CAR^WT^ T cells after 40 days after infusion). Mechanistically, recyclable CAR T cells contain more signaling endosomes, which amplify 4-1BB signaling and facilitate the metabolic reprogramming of the CAR T cells toward oxidative phosphorylation, which could explain the enhanced memory T-cell development and the improved *in vivo* persistence of recyclable CAR T cells. Overall, our study demonstrated that the recyclable CAR T cells show a superior antitumor activity, which may offer a straightforward and universal solution to enhance the persistence and functionality of CAR T cells containing the 4-1BB signaling domain.

**Table 1:** Summary of recyclable CAR’s features.

Features	Wild-type CAR	Recyclable CAR
CAR ubiquitination	Yes	No
CAR lysosomal degradation	Yes	No
CAR half-life	Shorter (~6 h)	Longer (≫9 h)
Long-term killing	*	***
*In vivo* persistence (BBζ CAR)	*	*** (100-fold greater on Day 40)

Asterisk(s) indicate the strength of the feature.

There are still several questions about CAR ubiquitination remaining to be addressed. First, what form of ubiquitination occurs on CAR proteins, poly-ubiquitination or multiple mono-ubiquitination? If it is poly-ubiquitination, what types of ubiquitin linkage are involved? Second, which E3 ligase, the enzyme that recognizes the substrate and mediates the covalent linkage between ubiquitin and the substrate, mediates CAR ubiquitination? Could this E3 be a potential therapeutic target to enhance CAR T therapy efficacy? Third, we showed that 4-1BB CAR signaling can be propagated in endosomes and the recyclable CAR design significantly increases the number of signaling endosomes. How endosomal CAR signaling is regulated will be an interesting question for future investigation, which may help optimizing the CAR T function.
